# EHR based Genetic Testing Knowledge Base (iGTKB) Development

**DOI:** 10.1186/1472-6947-15-S4-S3

**Published:** 2015-11-25

**Authors:** Qian Zhu, Hongfang Liu, Christopher G Chute, Matthew Ferber

**Affiliations:** 1 Department of Information Systems, University of Maryland Baltimore County, Baltimore, MD, USA; 2 Department of Health Sciences Research, Mayo Clinic, Rochester, MN, USA; 3 Division of General Internal Medicine, Johns Hopkins University, Baltimore, MD, USA; 4 Department of Laboratory Medicine and Pathology, Mayo Clinic, Rochester, MN, USA

**Keywords:** Clinical feature, Genetic disorder, Genetic test, EHR, iGTKB

## Abstract

**Background:**

The gap between a large growing number of genetic tests and a suboptimal clinical workflow of incorporating these tests into regular clinical practice poses barriers to effective reliance on advanced genetic technologies to improve quality of healthcare. A promising solution to fill this gap is to develop an intelligent genetic test recommendation system that not only can provide a comprehensive view of genetic tests as education resources, but also can recommend the most appropriate genetic tests to patients based on clinical evidence. In this study, we developed an EHR based Genetic Testing Knowledge Base for Individualized Medicine (iGTKB).

**Methods:**

We extracted genetic testing information and patient medical records from EHR systems at Mayo Clinic. Clinical features have been semi-automatically annotated from the clinical notes by applying a Natural Language Processing (NLP) tool, MedTagger suite. To prioritize clinical features for each genetic test, we compared odds ratio across four population groups. Genetic tests, genetic disorders and clinical features with their odds ratios have been applied to establish iGTKB, which is to be integrated into the Genetic Testing Ontology (GTO).

**Results:**

Overall, there are five genetic tests operated with sample size greater than 100 in 2013 at Mayo Clinic. A total of 1,450 patients who was tested by one of the five genetic tests have been selected. We assembled 243 clinical features from the Human Phenotype Ontology (HPO) for these five genetic tests. There are 60 clinical features with at least one mention in clinical notes of patients taking the test. Twenty-eight clinical features with high odds ratio (greater than 1) have been selected as dominant features and deposited into iGTKB with their associated information about genetic tests and genetic disorders.

**Conclusions:**

In this study, we developed an EHR based genetic testing knowledge base, iGTKB. iGTKB will be integrated into the GTO by providing relevant clinical evidence, and ultimately to support development of genetic testing recommendation system, iGenetics.

## Introduction

Individualized medicine, as a rapidly advancing field of healthcare, intends to enable accurate predictions about a person's susceptibility of developing disease, the course of disease, and its response to treatment based on genetic, genomic, and clinical information of individual patients. [1-5] No wonder so much hope is riding on the promise of "individualized medicine", particularly genetic screening and other tests provide more confident evidence for tailoring treatments to patients, potentially improving health care and saving money. With the recent advances in genetic technology, genetic tests are performed by over 500 laboratories for over 2,000 rare and common medical conditions. [6] These tests can effectively help health professionals determine or predict the genetic conditions for their patients. However, physicians have not actively incorporated these tests into their clinical practices partly due to the lack of the familiarity and supportive evidence of those genetic tests according to two recent national surveys commissioned by UnitedHealth Group in conjunction with Harris Interactive (n = 2,760; fieldwork conducted in January and February 2012). [7] Obviously, there is an urgent need to develop an intelligent system that will provide necessary information and guidance to assist physicians in applying genetic tests in their regular clinical practices. Ideally, this system will be able to 1) provide comprehensive information about genetic tests as education resources; 2) recommend the most appropriate genetic tests to patients based on clinical evidence.

Since the inception of the Human Genome Project [8] in 1990, a large portion of genetic testing information has been accumulated accordingly. The Clinical Pharmacogenetics Implementation Consortium (CPIC) [9] published pharmacogenomics guidelines in peer reviewed journals. [10-19] NIH maintains a list of genetic testing relevant data resources including GTR (Genetic Testing Registry), [9] ClinVar, [20] MedGen. [20] Electronic health records (EHR) include a wide spectrum of clinical information about patients, such as medical history, laboratory tests including genetic tests. Particularly, EHR data has attracted much more interests in accelerating individualized medicine research, [21,22] given a systematic collection of health information contained in EHR systems. [23] For instance, the NHGRI-funded eMERGE network (electronic Medical Records and GEnomics), [24] is coupling DNA biobanks to large comprehensive EHRs (containing millions of patients) for large-scale, high-throughput genetic research with the ultimate goal of returning genomic testing results to patients in a clinical care setting. To our knowledge, no efforts have been made to extract clinical evidence regarding to genetic testing from EHR to support genetic test recommendation. In this paper, we introduce our contribution in this particular area.

We have developed GTO (Genetic Testing Ontology) [25] by integrating GTR, ClinVar, HPO (Human Phenotype Ontology) [26] as well as scientific evidence extracted from the SemMedDB. [27] The capability of providing sufficient information regarding to particular genetic diseases or genetic tests and recommending appropriate genetic tests based on the clinical observation and professional knowledge, has been demonstrated in our previous study. [25] To enhance the aforementioned capability of the GTO with more concrete clinical evidence identified from EHR systems, in this study, we extracted and determined dominant clinical evidence corresponding to genetic tests operated at Mayo Clinic in 2013. Those clinical evidence will be integrated into the GTO. Information about genetic tests available in the EHR systems at Mayo Clinic is in semi-structured format, we utilized a Natural Language Processing (NLP) suite, MedTagger to extract information from the EHR and statistical analysis has been performed to determine the most relevant clinical features accordingly for each test. More detail about clinical evidence extraction is described in the Background and Methods section. Pros and cons about this study has been versioned and discussed in discussion section.

## Background and methods

### Genetic testing operation at Mayo Clinic

Genetic testing is a type of medical test to analyze chromosomes, genes, or proteins. The results of a genetic test can detect suspected heritable medical condition and furthermore determine the percentage of developing or passing on a genetic disorder for individual patient. More than 1,000 genetic tests are currently in use, and more are being developed.

**Figure 1:**
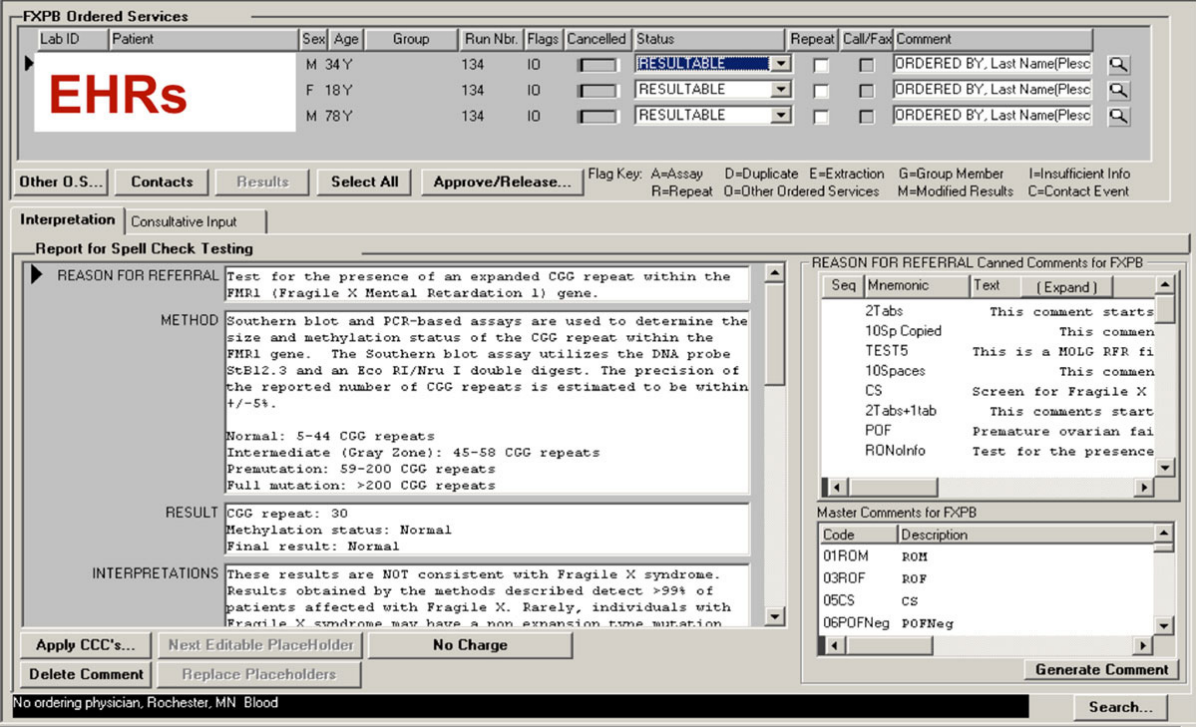
**Genetic test template available at the DLMP**.

At Mayo Clinic, there are about 65,000 samples being tested per year. Among these 65,000 samples, about 3,250 samples (5%) are from Mayo Clinic, and the rest of samples are referred from outside Mayo Clinic. In this study, we focused on the 5% Mayo Clinic patients, as their complete medical records are available in the EHR systems. The Department of Laboratory Medicine and Pathology (DLMP) at Mayo Clinic maintains genetic testing information that include patient clinical id, date of birth, gender, test reported, collected and received date, reason for referring, test results and interpretation, shown in Figure 1. We had an IRB approval (13-008995) to allow us accessing patient data for this study.

### Clinical evidence extraction

In this retrospective study, we analyzed patients' medical information extracted from the EHRs in order to determine the most significant clinical features observed in particular population groups for each genetic test. Four steps performed accordingly, are described as below.

#### Genetic test selection

In this study, we obtained information about 61 genetic tests operated at Mayo Clinic in 2013. In order to access patients' complete lists of their medical history, only patients from Mayo Clinic were included in this study. In addition to ensure accuracy of data analysis with enough samples, we mannually selected 7 genetic tests being tested for more than 100 patients. However, there are two genetic tests are used to detect risk for multiple genetic disorders respectively. "BRAF Mutation Analysis (V600E) Tumor" with internal Mayo test id "87980" is used to detect BRAF V600E mutation for diagnosis of Lung adenocarcinoma, Colorectal adenocarcinoma, Brain glioma, and etc. "Hereditary Nonpolyposis Colorectal Cancer (HNPCC) Screen" with Mayo internal test id "82500" is used to evaluate tumor tissue for evidence of defective DNA mismatch repair and consequently support further diagnosis of Gallbladder adenocarcinoma, Brain astrocytoma, Colorectal adenocarcinoma and etc. We excluded these two tests due to a small sample size resulted for each involved genetic disorder accordingly. Thus, total five genetic tests were selected for this study, and listed in Table 1.

**Table 1:** Information about five selected genetic tests

Test ID	Genetic test Name	Genetic Disorder
82993	Alpha-1 Antitrypsin	Alpha 1-antitrypsin deficiency

81508	Hemochromatosis HFE Gene Analysis, Blood	Hereditary hemochromatosis

61247	EGFR Gene. Mutation Analysis	Lung Cancer

9497	Cystic Fibrosis Mutation Analysis, 106 Mutation Panel	Cystic Fibrosis

9569	Fragile X Syndrome, Molecular Analysis	Fragile X Syndrome

#### Patient group composition

To compare and determine the most relevant clinical features for each genetic disorder based on the patients' medical history, we composed four different population groups. Group A (All) is consisted of 138, 229 patients from Mayo Clinic Employee Community Health (ECH); group T (Tested) is consisted of Mayo patients being tested by one of five selected genetic tests accordingly; group P (Positive) is consisted of Mayo patients being tested as positive for the genetic test; and group N (Negative) is consisted of Mayo patients being tested as negative for the genetic test. Genetic testing information is stored in semi-structured format, for instance, patient general information including patient id, sex, date of birth is in structured format, but referring reason, test results, and interpretation are in free text. To our knowledge, there is no NLP tool for extracting test result and observation for genetic tests, we manually reviewed the test results and separated test samples into group P and group N. Specifically, each test includes predefined categories to desribe specific "test result" and relevant "interpretation". For example, categories for "test result" of the test "9497" are summarized in Table 2. We manully reviewed such category information assigned to each patient for each test, and sepreated group T to group P and group N.

**Table 2:** Categories of test results for test "9497"

• NO CHARGE Test canceled, ordered in duplicate with order number 6606002374.
• None of the listed mutations were detected, indicating a revised risk of 1/507 (see interpretation). Intron 8 poly T alleles are 7T/7T.
• None of the listed mutations were detected.
• One copy each of the delta F508 and A349V mutation in exons 10 and 7 respectively, was identified.
• One copy each of the deltaF508 and 3905insT mutations in exons 10 and 20 respectively, was identified.
• One copy of the R117C mutation in exon 4 was identified.
• One copy of the R117H mutation in exon 4 was identified. Intron 8 poly T alleles are 7T/7T.
• One copy of the R334W mutation in exon 7 was identified.
• One copy of the R553X mutation in exon 11 was identified.
• One copy of the deletion of exons 2-3 was identified.
• One copy of the deltaF508 mutation in exon 10 was identified.
• Test canceled. Testing already performed on the patient in past thus testing is canceled per genetic counselor Vickie. NO CHARGE
• This is a testing accession
• Two copies of the S549R(T>G) mutation in exon 11 were identified.
• Two copies of the deltaF508 mutation in exon 10 were identified.

#### Clinical feature extraction

In order to extract clinical feature from clinical notes more intentionally, avoiding non-relevant clinical features interfering the final annotation results and decreasing the computation cost, we first collected the most common clinical features from a local copy of the HPO for each genetic disorder. In order to facilitate further data integration, we mapped all collected clinical features to Unified Medical Language System (UMLS) via UMLS MetMap API. Then for each genetic test, we extracted mentions of the identified clinical features from clinical notes for each patient group by applying an existing NLP tool, MedTagger suite. [28,29]

#### Clinical feature prioritization

For each genetic test, we computed frequency of each clinical feature occurring in the above four patient groups by using Eq.(1), and we manually compared the calculated frequencies across four groups for each clinical feature. The frequency of each clinical feature in increasing trend among the four population groups, from group A to group N, has been labeled as an enriched clinical feature. In addition to further quantitively measure the significance of the enriched clinical features impacting genetic test results, we calcualted odds ratio between group P and group N, as it is critical to determine the significance of the features by assessing odds and probability of clinical features identified among patients with positive test results against negative test results.

(1) Feature% = Number of patients with the clinical feature / Total number of patients in that group

## Results

### Data collection for genetic tests

• *Genetic testing information*. We obtained 3,595 patients' genetic testing information for 61 tests. As mentioned before, only five tests with sample size greater than 100 have been selected for this study. Thus, 2,054 patients being tested by the five selected genetic tests were remained in this study. Of these 2,054 patients, 604 patients without clinical notes have been excluded. Total 1,450 patients with test information including clinical id, date of birth, gender, reason for referring, test results, interpretation and their medical records have been extracted for further data analysis.

• *Patient cohort*. Four patient groups have been composed to compare and determine the significance of clinical features. Group A including 138,229 patients from the ECH was retrieved as a reference patient group to provide overall prevalence level of the clinical features for each genetic test. For each test, by excluding the patients whose test was cancelled after the test been prescribed and inserted into the DLMP system, the remaining patients are consisted of group T. By manully reviewing category information of "test results" for each test, we sepreated group T to group P and group N. The distribution of these four patient groups is shown in Table 3.

**Table 3:** Genetic testing information

Test ID	# of patients in group T	# of patients in group P	# of patients in group N
82993	514	70	464

81508	427	187	240

61247	101	15	86

9497	220	18	202

9569	168	9	159

### Clinical feature collection

In this study, we programmatically identified 243 common clinical features for five genetic disorders from the HPO. We applied MedTagger to annotate mentions of the identified clinical features in clinical notes of study subjects. We excluded the clinical features that are mentioned in less than one patient's clinical notes in the group P and group N. Thus, the remaining 60 clinical features were left for further analysis. It is worthy to note that there is no mention of any of the identified clinical features in clinical notes for tested patients for the test "61247", thus we excluded this test for further analysis. Four genetic tests along with the number of clinical features from the original list and annotation results are listed in Table 4.

**Table 4:** Distribution of clinical features

Test ID	# of CFs selected from the HPO	# of CFs mentioned in the clinical notes
82993	9	6

81508	86	38

61247	4	0

9497	74	7

9569	131	9

### Clinical feature prioritization

We calculated and compared numbers of patients and frequencies of each clinical feature among four population groups. In Table 5 enriched clinical features in bold are manually selected based on frequency comparison across four population groups. Subsequently we calculated odds ratio for each clinical feature by comparing group P and group N, the results are listed in Table 6. The clinical features with odds ratio greater than 1 were selected as dominant features and shown in bold in Table 6. For example, "hepatic failure", "emphysema" are the two clinical features with high odds ratio (greater than 1) for test "82993". Patients with these two clinical features will be highly recommended for the genetic test, "Alpha-1 Antitrypsin". The clinical features with their odds ratio as ranking weight, along with their associated genetic tests and genetic disorders have been loaded into iGTKB for further data integration to the GTO and genetic testing recommendation.

**Table 5:** Statistical numbers for clinical features identified from patients' clinical notes

Test id	Clinical Features	#Pat_A	#Pat_T	#Pat_N	#Pat_P	Freq_A	Freq_T	FreqN	FreqP
9497	**Malabsorption**	1036	3	1	2	0.00749	0.01364	0.00495	0.11111

9497	**Emphysema**	1058	2	1	1	0.00765	0.00909	0.00495	0.05556

9497	**Chronic bronchitis**	920	3	2	1	0.00666	0.01364	0.00990	0.05556

9497	**Bronchiectasis**	350	40	36	4	0.00253	0.18182	0.17822	0.22222

9497	**Anemia**	10832	23	21	2	0.07836	0.10455	0.10396	0.11111

9497	Diarrhea	26264	29	27	2	0.19000	0.13182	0.13366	0.11111

9497	Asthma	29676	50	47	3	0.21469	0.22727	0.23267	0.16667

9569	**Tremor at rest**	3093	8	6	2	0.02238	0.04762	0.03774	0.22222

9569	**Joint hypermobility**	48	4	3	1	0.00035	0.02381	0.01887	0.11111

9569	**Moderate mental retardation**	500	4	3	1	0.00362	0.02381	0.01887	0.11111

9569	**Otitis media**	11064	8	7	1	0.08004	0.04762	0.04403	0.11111

9569	**Intellectual disability, moderate**	28	11	10	1	0.00020	0.06548	0.06289	0.11111

9569	**Depression**	42371	35	33	2	0.30653	0.20833	0.20755	0.22222

9569	Seizure	14498	47	46	1	0.10488	0.27976	0.28931	0.11111

9569	Seizures	14498	47	46	1	0.10488	0.27976	0.28931	0.11111

9569	Anxiety	36674	47	46	1	0.26531	0.27976	0.28931	0.11111

81508	**Telangiectasia**	1340	7	1	6	0.00969	0.01639	0.00417	0.03209

81508	**Autosomal dominant**	216	6	2	4	0.00156	0.01405	0.00833	0.02139

81508	**Autosomal dominant inheritance**	216	6	2	4	0.00156	0.01405	0.00833	0.02139

81508	**Skin hyperpigmentation**	25	3	1	2	0.00018	0.00703	0.00417	0.01070

81508	**Hepatic fibrosis**	113	3	1	2	0.00082	0.00703	0.00417	0.01070

81508	**Hypogonadotrophic hypogonadism**	767	3	1	2	0.00555	0.00703	0.00417	0.01070

81508	**Hypogonadism, hypogonadotropic**	733	3	1	2	0.00530	0.00703	0.00417	0.01070

81508	**Generalized hyperpigmentation**	651	10	4	6	0.00471	0.02342	0.01667	0.03209

81508	**Abnormal bleeding**	2396	15	6	9	0.01733	0.03513	0.02500	0.04813

81508	**Bleeding tendency**	9682	11	5	6	0.07004	0.02576	0.02083	0.03209

81508	**Heart failure**	3009	20	10	10	0.02177	0.04684	0.04167	0.05348

81508	**Enlarged liver**	25	2	1	1	0.00018	0.00468	0.00417	0.00535

81508	Osteoporosis	23640	67	34	33	0.17102	0.15691	0.14167	0.17647

81508	Generalized osteoporosis	23640	67	34	33	0.17102	0.15691	0.14167	0.17647

81508	**Arrhythmia**	2362	15	8	7	0.01709	0.03513	0.03333	0.03743

81508	Erectile dysfunction	4418	21	12	9	0.03196	0.04918	0.05000	0.04813

81508	Abdominal pain	37984	96	57	39	0.27479	0.22482	0.23750	0.20856

81508	Alopecia	1243	5	3	2	0.00899	0.01171	0.01250	0.01070

81508	Arthralgia	17292	53	32	21	0.12510	0.12412	0.13333	0.11230

81508	Joint pain	16232	48	29	19	0.11743	0.11241	0.12083	0.10160

81508	Cardiomyopathy	1220	13	8	5	0.00883	0.03044	0.03333	0.02674

81508	Hepatic cirrhosis	62	13	8	5	0.00045	0.03044	0.03333	0.02674

81508	Cirrhosis	415	173	108	65	0.00300	0.40515	0.45000	0.34759

81508	Hepatomegaly	244	24	15	9	0.00177	0.05621	0.06250	0.04813

81508	Cardiomegaly	1751	11	7	4	0.01267	0.02576	0.02917	0.02139

81508	Diabetes mellitus	9692	64	41	23	0.07012	0.14988	0.17083	0.12299

81508	Congestive heart failure	2562	14	9	5	0.01853	0.03279	0.03750	0.02674

81508	Hepatocellular carcinoma	85	52	34	18	0.00061	0.12178	0.14167	0.09626

81508	Ascites	327	141	94	47	0.00237	0.33021	0.39167	0.25134

81508	Cholestasis	113	9	6	3	0.00082	0.02108	0.02500	0.01604

81508	Hepatic failure	21	9	6	3	0.00015	0.02108	0.02500	0.01604

81508	Hypoglycaemia	4500	32	23	9	0.03255	0.07494	0.09583	0.04813

81508	Hypoglycemia	4500	32	23	9	0.03255	0.07494	0.09583	0.04813

81508	Liver failure	244	41	30	11	0.00177	0.09602	0.12500	0.05882

81508	Splenomegaly	307	46	36	10	0.00222	0.10773	0.15000	0.05348

81508	Rapidly progressive	2167	7	6	1	0.01568	0.01639	0.02500	0.00535

81508	Rapid progression	2128	7	6	1	0.01539	0.01639	0.02500	0.00535

81508	Pleural effusion	510	10	9	1	0.00369	0.02342	0.03750	0.00535

82993	**Emphysema**	1058	16	12	4	0.00765	0.03113	0.02586	0.05714

82993	**Hepatic failure**	21	7	6	1	0.00015	0.01362	0.01293	0.01429

82993	Cirrhosis	415	160	142	18	0.00300	0.31128	0.30603	0.25714

82993	Chronic obstructive pulmonary disease	2048	18	16	2	0.01482	0.03502	0.03448	0.02857

82993	Hepatocellular carcinoma	85	53	48	5	0.00061	0.10311	0.10345	0.07143

82993	Hepatomegaly	235	24	22	2	0.00170	0.04669	0.04741	0.02857


# Pat_A: number of patients in group A have the clinical feature accordingly; # Pat_T: number of patients in group T have the clinical feature accordingly; # Pat_P: number of patients in group P have the clinical feature accordingly; # Pat_N: number of patients in group N have the clinical feature accordingly; Freq_A: frequency of the clinical feature occurring in group A; Freq_T: frequency of the clinical feature occurring in group T; Freq_P: frequency of the clinical feature occurring in group p; Freq_N: frequency of the clinical feature occurring in group N.

**Table 6:** Statistical analysis results

Test ID	Clinical Features	OR
9497	**Bronchiectasis**	1.31746

9497	Asthma	0.65957

9497	Diarrhea	0.81019

9497	**Anemia**	1.07738

9497	**Malabsorption**	25.12500

9497	**Chronic bronchitis**	5.88235

9497	**Emphysema**	11.82353

9569	**Depression**	1.09091

9569	**Tremor at rest**	7.28571

9569	Seizure	0.30707

9569	Seizures	0.30707

9569	Anxiety	0.30707

9569	**Intellectual disability, moderate**	1.86250

9569	**Otitis media**	2.71429

9569	**Joint hypermobility**	6.50000

9569	**Moderate mental retardation**	6.50000

81508	**Telangiectasia**	7.92265

81508	**Autosomal dominant**	2.60109

81508	**Autosomal dominant inheritance**	2.60109

81508	**Skin hyperpigmentation**	2.58378

81508	**Hepatic fibrosis**	2.58378

81508	**Hypogonadotrophic hypogonadism**	2.58378

81508	**Hypogonadism, hypogonadotropic**	2.58378

81508	**Generalized hyperpigmentation**	1.95580

81508	**Abnormal bleeding**	1.97191

81508	**Bleeding tendency**	1.55801

81508	**Heart failure**	1.29944

81508	**Enlarged liver**	1.28495

81508	**Osteoporosis**	1.29832

81508	**Generalized osteoporosis**	1.29832

81508	**Arrhythmia**	1.12778

81508	Erectile dysfunction	0.96067

81508	Abdominal pain	0.84602

81508	Alopecia	0.85405

81508	Arthralgia	0.82229

81508	Joint pain	0.82287

81508	Cardiomyopathy	0.79670

81508	Hepatic cirrhosis	0.79670

81508	Cirrhosis	0.65118

81508	Hepatomegaly	0.75843

81508	Cardiomegaly	0.72756

81508	Diabetes mellitus	0.68070

81508	Congestive heart failure	0.70513

81508	Hepatocellular carcinoma	0.64532

81508	Ascites	0.52143

81508	Cholestasis	0.63587

81508	Hepatic failure	0.63587

81508	Hypoglycaemia	0.47704

81508	Hypoglycemia	0.47704

81508	Liver failure	0.43750

81508	Splenomegaly	0.32015

81508	Rapidly progressive	0.20968

81508	Rapid progression	0.20968

81508	Pleural effusion	0.13799

82993	Cirrhosis	0.78494

82993	Hepatocellular carcinoma	0.66667

82993	**Emphysema**	2.28283

82993	Hepatomegaly	0.59091

82993	Chronic obstructive pulmonary disease	0.82353

82993	**Hepatic failure**	1.10628


OR: Odds Ratio

## Discussion

Genetic testing allows genetic diagnosis of vulnerabilities to inherited diseases, and determine a child's parentage (genetic mother and father) or in general a person's ancestry. However, insufficient resources and tooling hinder incorporating genetic testing into regular clinical practice. In this paper, we introduce an EHR based genetic testing knowledge base, iGTKB, as a fundamental clinical evidence resource in computational manor that will be able to better assist clinical decision making, especially to support development of an individualized genetic test recommendation system, iGenetics. In this section, we discuss multiple benefits gained as well as challenges and issues arising from this preliminary work and proposed future plans accordingly.

### A. An executable genetic testing resource, iGTKB

Multiple resources, such as GTR, ClinVar, GeneReview maintained by the NIH contain comprehensive genetic testing information, which is browseable through NIH websites. In the meantime, authorized professionals are manually defining and approving genetic testing guidelines as golden standard to guide physicians to prescribe appropriate genetic tests. For instance, CPIC [9] as a shared project between PharmGKB [30] and the PGRN [31] publishes pharmacogenomics guidelines that are peer-reviewed and published in a leading journal. The goal of the CPIC is to publish 3-4 guidelines per year as this is an entire manual curation process. More and more efforts have been/will be made to provide and maintain new and the existing resources, as genetic testing is one of critical steps towards individualized medicine. However, most of these existing resources are primarily based on domain knowledge and information provided by the laboratories. To our knowledge, there is no effort made by mining EHR data to identify individualized information about genetic testing from patient perspective. In this study, we focused on genetic testing information and patient information retrieved from the EHR systems at Mayo Clinic and successfully identified dominant clinical features for the selected genetic tests. In our previous study, we have generated a genetic testing ontology (GTO) by integrating GTR, ClinVar, HPO and SemMedDB. In the next step, we will integrate clinical evidence identified from this study and our previous study [32] into the GTO to provide more comprehensive view of genetic test and ultimately support automation of genetic test recommendation.

### B. Clinical feature identification

Clinical features are key clinical factors to support clinical decision making, particularly to guide genetic test prescription. Thus it is very important to accurately and completely identify a list of clinical features as dominate clinical factors for each genetic disease. Therefore, in this study, two steps have been performed to identify clinical features from the EHR data. 1) For each genetic disorder, we automatically searched the HPO for clinical features accordingly, which were used to direct annotation with clinical notes in free text. 2) We systematically annotated clinical notes to extract mentions of the clinical features identified in the first step, and statistically quantified significance of individual clinical features to corresponding genetic tests by calculating odds ratio. The strategy of clinical feature identification is effectively identify the most common clinical features and dramatically decrease the computation cost as no extraction performed for those non-relevant features.

Based on the current experiment, some of the clinical features have zero occurance in the EHR data. The main reason causing such absense is the identified clinical features from the HPO have high level descriptions, such as "abnormal head movement". However, "abnormal head movement" with zero occurrence does not mean that there is no patient with abnormal head movement. It is just because physicians do not describe in this way in the clinical notes. Deep phenotyping will be proposed for more in-depth SME involvement.

In this study, MedTagger has been applied to extract clinical features from patients' clinical notes only. The reason why we skipped to annotate information presented in family history is that family history is not always documented in clinical notes at Mayo Clinic. To avoid false positive results generated from statistical analysis due to inconsistent occurrence of family history and patient's medical history in clinical notes in this early stage of experiment, we excluded annotation for family history. To avoid such shortcoming occurring in the EHR data, literature based analysis to identify information about family history will be one of the solutions. Previously we have conducted a preliminary study [33] to analyze 10 randomly selected chapters of GeneReviews, [33] which is "expert-authored, peer-reviewed disease descriptions ("chapters") presented in a standardized format and focused on clinically relevant and medically actionable information on the diagnosis, management, and genetic counseling of patients and families with specific inherited conditions". Family history as one of sections is included in each chapter of GeneReviews. In the future study, we will integrate annotation results including information about family history from GeneReviews to the GTO, to provide extra criteria for genetic testing guideline generation.

In this study, we were focusing on sign and symptoms mentioned in clinical notes as clinical features. However, there are several other clinical characteristics mentioned in the report of radiology, laboratory and/or medications, which also provide comprehensive information for genetic testing guideline. We will extract and integrate that information into the iGTKB for supporting accurate genetic test predictions.

### C. Clinical feature prioritization

In this study, the patient sample size of each genetic test is still relatively small as only information about tests operated in 2013 at Mayo Clinic has been included. Small sample size resulted in insufficient annotation results produced consequently, which significantly impacted our statistical analysis for clinical feature prioritization. In the next step, we will request more data from the DLMP, especially increasing the time window of test operation to include more eligible genetic tests.

Two steps have been performed to prioritize clinical features for each genetic test accordingly, manual enrichment analysis and statistical analysis based on odds ratio, which produced consistent results, shown in Table 5 and Table 6. The clinical features with high odds ratio illustrate their significance to the genetic tests, as odds ratio was calculated between group P and group N. However, for those with lower odds ratio maybe also meaningful clinical features in clinical settings based on their overall prevalence distribution. For example, "cirrhosis" as one of clinical features for "alpha-1 antitrypsin deficiency", corresponding to the test "82993", odds ratio is 0.78494, less than 1, however, the odds ratio calculated based on group T and group A to reflect overall prevalence distribution is 150.09353, the highest value comparing to other features. The reason for such huge difference between these two types of odds ratio is this clinical feature had been observed in a large number of patients from group N (patients with negative test result). To this end, "cirrhosis" has been considered as one of key clinical features for test prescription, although a majority of patients have negative test results accordingly. Thus, to avoid losing any possible signals, we will integrate clinical features with odds ratio calculated based on group P and group N, as well as prevalence ratio calculated based on group T and group A as one reference criteria into the GTO.

### D. Genetic testing result annotation

As mentioned before, there are no NLP tools available for genetic testing information extraction and normalization. Currently we manually reviewed genetic testing results as well as test result interpretation to separate group T into group P and group N. However, manual review is still cumbersome. Thus, we will work with DLMP to seek possibility that we can help them to define a normalized genetic test registry template. Ideally the template will include normalized terms to indicate test results and interpretation that can be parsed programmatically, ultimately, it will facilitate the adoption of such useful information for research purpose. Consequently, development of an NLP tool for automated genetic testing information extraction is necessary to accelerate the pace of building the iGTKB and integrating more genetic testing information programmatically, which will be the next step.

### E. Patient cohort identification based on ICD9

**Figure 2:**
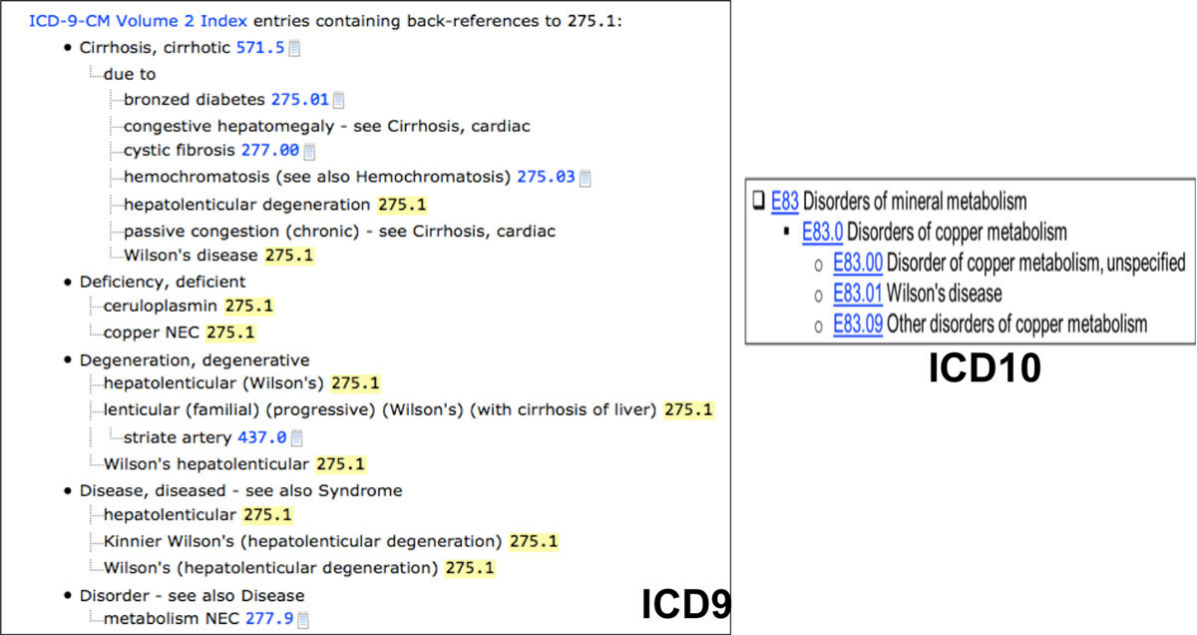
**ICD9 and ICD10 definition for WD**.

In our previous study, we extracted group D namely a patient group consisting of patients based on their ICD 9 codes. However, the accuracy and completeness of patient retrieval for this group is very low due to the natural structure of ICD 9, comparing to the refined definition of ICD 10. For example, one ICD 9 code, "273.5" has been defined and used to label multiple disorders including Wilson's disease. In comparison, "E83.01", an ICD 10 code for Wilson's disease is a child node of "Disorders of copper metabolism" (E83.0), which is shown in Figure 2. This resulted that the number of retrieved patients according to the ICD 9 codes of genetic disorders is very small. For example, we have extracted 240 patients have been tested for "Cystic Fibrosis Mutation Analysis" however, the total number of patients we retrieved based on the ICD 9 codes "277.00", "277.01", "277.02", "277.03" and "277.09" of "Cystic Fibrosis" is only 8. Such inaccurate and incomplete list of patients identified based on ICD 9 has negative impact on further analysis. Thus, in this study, we excluded group D based on the ICD9 codes. Given the importance of group D, alternatively, we propose two steps to further improve patient retrieval based on their diagnosis in the next step, 1) use both of codes, ICD 9 and ICD 10 if possible (ICD 10 is rarely being used in current EHR systems) for patient cohort retrieval; 2) apply more concrete phenotype algorithms for patient cohort retrieval. Those algorithms will not only rely on the diagnosis codes, but also medications and appropriate laboratory test will be considered to determine patients with a particular disease. Some algorithms can be found at PheKB [34], or generated with help from the domain experts.

### F. Genetic test recommendation

This is a first step to generate an EHR based genetic testing knowledge base, and test recommending signals have been shown based on existing evidence available in the iGTKB. However, it can not support test prediction for patients with clinical features that are not documented in the iGTKB. Such functionality will be designed and embedded into iGenetics, which is developed on top of the next generation of the GTO with other resources we generated previously. [25,32,33]

## Conclusion

In this study, we successfully built an EHR based genetic testing knowledge base (iGTKB). The current version of iGTKB consists information regarding to genetic test as well as relevant genetic disorders, and clinical features extracted from the EHR systems. To enlarge its recommendation capability, we will integrate iGTKB and information extracted from the GeneReviews into the GTO to generate a comprehensive computational genetic testing resource for supporting iGenetics development.

## Competing interests

The authors declare that they have no competing interests.

## Authors' contributions

QZ carried out the experiment and data analysis, and drafted the manuscript; HL exacted EHR data from Mayo Clinic EHR system. CGC: reviewed the manuscript and provided feedback and suggestions. MF helped genetic testing data retrieval. All authors have read and approved the final manuscript.

## Declarations

Publication costs for this article were funded by Mayo Center of Individualized Medicine.

This article has been published as part of *BMC Medical Informatics and Decision Making *Volume 15 Supplement 4, 2015: Selected articles from the IEEE International Conference on Bioinformatics and Biomedicine (BIBM 2014): Medical Informatics and Decision Making. The full contents of the supplement are available online at http://www.biomedcentral.com/bmcmedinformdecismak/supplements/15/S4.
